# Two Adults With Multisystem Inflammatory Syndrome Post-COVID-19 in a Lebanese Hospital: A Case Report

**DOI:** 10.7759/cureus.23440

**Published:** 2022-03-24

**Authors:** Amena Khatoon, Matina n Hamadeh, Zouheir Kreidly

**Affiliations:** 1 Infectious Diseases, Lebanese University Faculty of Medicine, Beirut, LBN; 2 Infectious Diseases, Sahel General Hospital, Beirut, LBN

**Keywords:** multi-system inflammatory disease in children (mis-c), sar-cov 2 infection, multisystem inflammatory syndrome in adults [mis-a], mis-a, covid 19

## Abstract

COVID-19 is a respiratory illness with multiple extra-pulmonary complications. The multisystem inflammatory syndrome (MIS) is one of its complications that usually affects children and is known as MIS-C, but several cases have been reported in adults, symbolized by MIS-A. Thus, the Centers for Disease Control and Prevention (CDC) developed a working case definition of MIS-A, which includes several criteria. Here we report two cases of adult male patients with clinical and laboratory symptoms consistent with MIS-A. Case one patient presented at a late stage after two weeks post the onset of symptoms. His health deteriorated rapidly, and eventually, he passed away. However, the second patient presented a few days after the symptoms’ onset and took a course of steroids. He was discharged home.

## Introduction

COVID-19 is a disease caused by SARS-CoV-2, and it results in respiratory illness, which can range from mild to severe [1]. Extra-pulmonary complications and sequelae of COVID-19 have been reported [2], and these mainly affect the cardiovascular system, central and peripheral nervous systems [2-3], besides negative psychological effects [2].

Multisystem inflammatory syndrome (MIS) in adults is an uncommon complication of COVID-19 [4]. The etiology of MIS-A is unknown, but it is suspected to be associated with direct endothelial damage due to delayed dysregulated immune complex activation or an antibody-mediated process [4-6]. Also, it is suggested to be triggered by the COVID-19 vaccine [7-8]. Salzaman et al. published in their article three cases of patients with a positive history of SARS-CoV-2 infection who developed MIS-A post-COVID-19 vaccination [7]. Also, Moriera J. reported in his article a case of MIS-A post vaccine and with negative history of COVID-19 [8]. However, Vogel T et al. noted in their article that this entity is still unknown, and there is a need to monitor this adverse event [9].

With respect to Lebanon, there are no published articles about MIS-A. Hence, we present two cases of MIS-A post-COVID-19 in a Lebanese hospital. 

## Case presentation

Case number one

Mr. R. was a 28-year-old previously healthy male patient who presented with a fever that started 18 days before presentation. It was accompanied by a sore throat for the first few days and then followed by several episodes of diarrhea. He also complained of myalgia, migratory arthralgia, and rash over the trunk and extremities, which was resolving spontaneously.

The patient sought medical advice as an outpatient, and his blood tests showed leukocytosis, a high C-reactive protein test, and erythrocyte sedimentation rate (ESR) (55mm/hr), slightly elevated liver function tests, and a negative Widal test. Polymerase chain reaction (PCR) of COVID-19 was done twice, and the results turned negative, so he was started on ciprofloxacin for a possible underlying infection. The patient didn’t improve and thus presented in the ED of our hospital. His initial vital signs in the emergency department (ED) were as follows: blood pressure of 100/65 mmHg, heart rate of 122 bpm, the temperature of 38.8°C, and normal oxygen saturation on room air (SPO2) of 99%. On physical exam, he looked ill and had a non-icteric sclera, non-purulent conjunctivitis, normal oral cavity, and no sinus tenderness. His chest was clear on auscultation, and he had normal heart sounds with tachycardia and a soft abdomen. The patient also had a mild rash over the trunk and extremities, which was resolving. He had no lower limbs edema or signs of arthritis. His initial abnormal laboratory results showed thrombocytopenia, acute kidney injury, and elevated liver function tests that worsened on day one (Table 1). Polymerase chain reaction (PCR) COVID-19 was negative in our hospital.

**Table 1:** Serial blood test results for Mr. R. WBC: white blood cell count; Lymph: lymphocytes; Plt: platelets; BUN: blood urea nitrogen; Creat: creatinine; SGPT: alanine aminotransferase; SGOT: aspartate aminotransferase; GGT: gamma-glutamyl transferase; AlkPh: alkaline phosphatase; Bil-T: total bilirubin; Bil-D: direct bilirubin; Trop: troponin; CRP: C-reactive protein; CPK: creatine phosphokinase; INR: international normalized ratio.

	Day 0	Day 1	Day 2
WBC	7.3k	4.1k	19k
Lymph	0.5k	0.5k	1.7k
Plt	62.6k	61.4k	96k
BUN	45	60	73
Creat	4.57	5.8	6.7
SGPT	358	516	702
SGOT		1358	3342
GGT	519	476	500
AlkPh		173	176
Bil-T	3.4		5.2
Bil-D	2.5		3.6
Trop ng/dl			1.9
CRP mg/dl	32		
CPK		1143	
INR		1.8	

**Figure 1:**
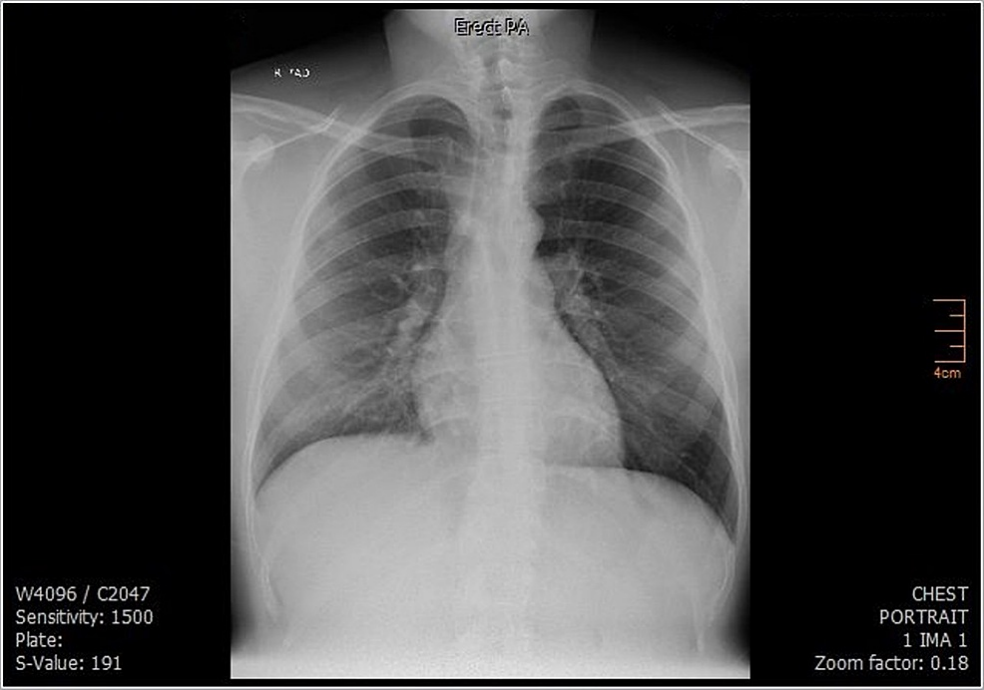
The first chest X-ray (CXR) of Mr. R. Chest X-ray done when Mr. R. presented to the emergency department, and it was normal.

An urgent blood smear was done at that time, and it was also normal. Chest X-ray (Figure 1) and ultrasound of the abdomen and pelvis were also normal. Two sets of blood cultures were ordered, in addition to Brucella titers and blocking antibodies, human immunodeficiency virus serology, hepatitis A virus serology, hepatitis C virus antibodies, Australia antigen, antinuclear antibody, antineutrophil cytoplasmic antibodies test, and ferritin.

**Figure 2:**
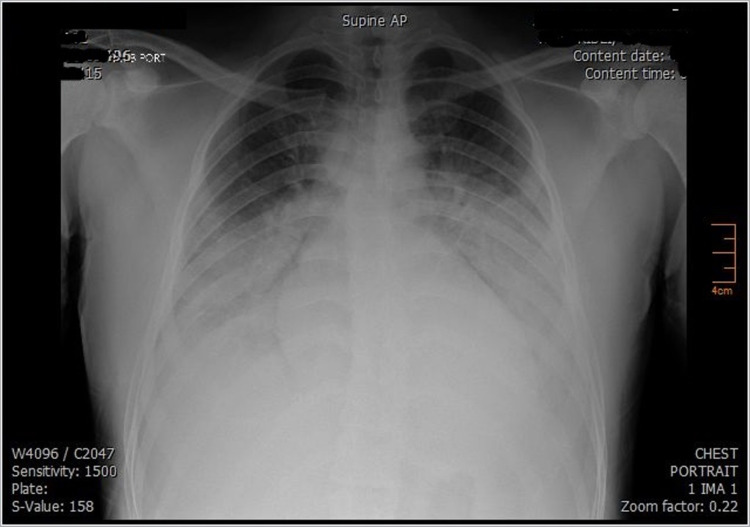
The second chest X-ray (CXR) of Mr. R. Second chest X-ray of Mr. R which was done after his health deteriorated. It showed bilateral pleural effusion and lung congestion suggestive of pulmonary edema.

The patient was started on intravenous hydration for possible prerenal azotemia. In the early morning of day two, he developed dyspnea and hypoxemia. Chest X-ray showed bilateral effusion and congestion suggestive of pulmonary edema (Figure 2). He was thus started on Lasix, and bilevel positive airway pressure (Bipap) was applied. However, the patient's health deteriorated rapidly within five hours, he was intubated, and then he developed ventricular fibrillation. Cardio-respiratory resuscitation was done for one and half hours, but the patient failed to respond and passed away. Bedside transthoracic echocardiography was done during the arrest, so wall motion abnormality could not be assessed, but cardiac tamponade was ruled out.

Pre-arrest were seen later in the blood tests, and the patient tested positive for COVID-19 serology with immunoglobulin M (IgM) 3.4 and immunoglobulin G (IgG) 4.2 (according to the hospital’s lab index: IgM considered negative if <1, and IgG negative if < 1.4). Blood cultures and hepatitis A virus antibodies were negative, but the other blood tests were not done, as financial clearance was awaited for them.

Case number two

Mr. A, a 40-year-old previously healthy male patient with a history of mild COVID-19 infection two months ago, presented with a history of fever and dyspnea for five days, followed by diarrhea, abdominal pain, and rash over the upper extremities, which were resolving spontaneously. Vital signs were as follows: heart rate of 127 bpm, blood pressure of 110/78 mmHg, and temperature of 38.5°C. His physical exam was normal except for a mild rash over the upper extremity that was improving. Initial blood test results showed elevated liver function tests only, and later his creatinine level rose, and the platelet count dropped; serial blood tests are listed in (Table 2).

**Table 2:** Serial blood test results of mr. A. WBC: white blood cell count; Lymph: lymphocytes; Plt: platelets; Creat: creatinine; BUN: blood urea nitrogen; SGPT: alanine aminotransferase; SGOT: aspartate aminotransferase; GGT: gamma-glutamyl transferase; AlkPh: Alkaline phosphatase; CRP: C-reactive protein; Trop: troponin.

	Day 0	Day 1	Day 2	Day 3	Day 4	Day 5	Day 6	Day 7	Day 8	Day 9
WBC	3.7k	2.8k	5.9k	10k	10.9k	8.5k			17.2k	13.6k
Lymph	0.3k	0.4k	2.2k	6.9k	5k	2.5k			3.2k	2.6k
Plt	196k	133k	180k	164k	236k	238k			529k	560k
Creat	1.19	1.73	1.01	0.8	0.78	0.86			0.75	
BUN	10	16			11					
SGPT	301	312	197	172	186	152			76	
SGOT	357	400	113	79	94	50			41	
GGT	342	302		259	288	244				
AlkPh	163	116		166	134	104				
Ferritin				3419			729			
D-dimer ng/dl				6038			1460			
CRP mg/dl	1.7	1.7	4.9		3.4	1.3				0.4
Trop ng/dl				0.014	0.007					

COVID-19 serology was positive for immunoglobulin G of 7.25 and negative for immunoglobulin M of 0.43. His chest X-ray was normal and blood cultures, Widal, and Weil-Felix tests turned back negative. Immunoglobulin M for Epstein-Barr virus, Cytomegalovirus, and hepatitis A virus was negative. Hepatitis C virus antibodies and Australian antigens were also negative.

He was admitted and started on intravenous steroids (Solu-Medrol 60mg every eight hours first five days then twice daily) and therapeutic dose low-molecular-weight-heparin (subcutaneous Lovenox 60mg twice daily). The patient’s symptoms improved gradually. On day two, the fever resolved, and he had no more dyspnea. However, he only complained of mild chest pain that remained for several days after starting treatment. Transthoracic echocardiography was done to rule out myocarditis and turned out to be normal. His blood tests also improved, and most of them returned to normal values by day nine. The patient was discharged on a tapering dose of steroids and oral anticoagulation.

## Discussion

Multisystem inflammatory syndrome in adults (MIS-A) should be considered in adult patients presenting with fever and extra-pulmonary symptoms but without severe respiratory illness. Besides, evidence of the recent history of SARS-CoV-2 infection, either by PCR or serology, should be present [10]. It usually occurs 2 to 12 weeks post SARS-CoV-2 infection [11].

According to the case definition made by CDC, it is an acute illness requiring hospital admission for ≥ 24 hours for a ≥ 21-year-old patient, or an illness resulting in death, with clinical and laboratory criteria and without an alternative diagnosis for this illness. The clinical criteria are divided into primary and secondary; it stands for fever ≥ 24 hours before admission or within the first three days of hospitalization and presences of ≥3 clinical criteria, with ≥1 being a primary one. The latter includes severe cardiac illness and rash with non-purulent conjunctivitis. While the secondary clinical criteria include new-onset neurological signs or symptoms, shock or hypotension, GI symptoms (abdominal pain, diarrhea, vomiting), and thrombocytopenia. In addition to clinical ones, laboratory evidence must be presented, and it includes elevation of at least two inflammatory markers (CRP, procalcitonin, ESR, ferritin, IL6), and positive COVID-19 PCR or serology tests [11-13]. 

Treatment should be started directly without delay, with steroids and/or intravenous immunoglobulin (IVIg), which are considered the mainstay of treatment of MIS-A according to Varyani et al.’s article [14].

Our patients had a fever for more than 24hrs, rash, thrombocytopenia, elevated liver function tests (LFTs) and creatinine, and positive COVID-19 serology tests, which are considered the main criteria for diagnosis of MIS-A [11-12].

Mr. R. was admitted at an advanced stage, and his health deteriorated very rapidly; he passed away on day two. According to the case definition mentioned above, the patient had: age > 21 years, fever for > 24 hours before hospitalization, severe illness resulting in death, two primary clinical criteria (rash with non-purulent conjunctivitis and ventricular dysfunction [v-fibrillation]), three secondary clinical criteria (lethargy, diarrhea, and thrombocytopenia), elevation in two inflammatory markers (CRP and ESR) and positive COVID-19 serology test. His latter blood test was seen after he passed away; hence, MIS-A was not in the differentials, especially since his three PCR COVID-19 tests that were done on different occasions were negative. Thus, MIS-A was diagnosed post-mortem.

However, Mr. A. had a known history of recent COVID-19 infection, making MIS-A top the list of differential diagnoses. Thus, intravenous steroids were directly started in addition to therapeutic doses of Lovenox, and the patient’s symptoms improved gradually along with his laboratory tests.

## Conclusions

Several complications of COVID-19 have been reported, including MIS-A that may occur after asymptomatic infection, and it could be fatal. Thus, physicians should be on the lookout for such a diagnosis and start the treatment immediately with steroids. We reported these cases of MIS-A to clarify the importance of early diagnosis that affects morbidity and mortality.
